# Surgical intervention may provides better outcomes for hip fracture in nonagenarian patients: A retrospective observational study

**DOI:** 10.1016/j.heliyon.2024.e25151

**Published:** 2024-01-26

**Authors:** Suo-Hsien Wang, Chia-Wei Chang, Shion-Wei Chai, Ting-Shuo Huang, Rueyshyang Soong, Ngi-Chiong Lau, Chih-Ying Chien

**Affiliations:** a Department of Surgery, New Taipei Municipal Tucheng Hospital, New Taipei City, 23652, Taiwan; b Department of Orthopaedic Surgery, Chang Gung Memorial Hospital, Keelung, Taiwan; c Department of General Surgery, Chang Gung Memorial Hospital, Keelung, Taiwan; d Department of General Surgery, Jen Ai Hospital, Taichung, 400, Taiwan; e School of Traditional Chinese Medicine, College of Medicine, Chang Gung University, Taoyuan, 333, Taiwan; f Division of Transplantation, Department of Surgery, Taipei Municipal Wan-Fang Hospital, Taiwan; g Institute of Emergency and Critical Care Medicine, National Yang Ming Chiao Tung University, Taiwan

**Keywords:** Nonagenarian, Hip fracture, Surgery

## Abstract

**Background:**

Hip fracture is a common disease in the elderly. Among these patients, surgical intervention for hip fracture should be carefully considered because of old age and multiple comorbidities. There are still insufficient comparisons between nonagenarian patients treated with surgery and those treated non-surgically. We studied hip fracture nonagenarian patients to compare the different outcomes between surgical and non-surgical treatments.

**Materials and methods:**

Nonagenarian patients visiting the emergency department with hip fractures between March 2010 and December 2020 were identified. Overall survival was estimated using multivariate Cox proportional hazards models. The mortality rates, the length of hospital stay, complication and readmission rates were also recorded.

**Results:**

A total of 173 patients who underwent surgery and 32 who received conservative treatments were included. The median survival time was 58.47 months in the OP group, which was significantly higher than the 24.28 months in the non-OP group. After adjusting for covariates, including age, sex, Charlson Comorbidity Index (CCI), injury severity score, and fracture type, the risk of death was reduced by surgery (hazard ratio [HR] = 0.427; 95 % confidence interval [CI]: 0.207–0.882; p = 0.021). CCI was also an independent risk factor for poor survival rate (HR = 1.3; 95 % CI: 1.115–1.515; p = 0.001). After adjusting for several factors, surgery within 48 h improved overall survival (HR: 2.518; 95 % CI: 1.299–4.879; p = 0.006) in operative group.

**Conclusion:**

Our study suggests that surgical treatment may provide better survival for nonagenarian patients with hip fractures than non-operation, especially patients with less concurrent comorbidities.

## 1 Introduction

Hip fractures are a major health care problem in developed countries. Ninety-five percent of elderly patients present with at least one comorbidity and numerous prescription medications [1]. The medical complexities associated with treating elderly patients lead to prolonged hospital stays, frequent readmissions, and a one-year mortality rate of approximately 25 % [[2], [3], [4], [5]]. As life expectancy around the world increases, diseases of the elderly population are also growing. Calculations expect the ongoing increase to reach 4.5 million hip fractures worldwide by 2050 [6]. In Taiwan, this trend is also high in both men and women aged over 85 years [7].

Management of hip fractures in the elderly is a challenging task with high morbidity and mortality in the population. Most deaths due to post-hip fracture are due to cardiovascular events and pneumonia with postoperative complications including delirium, pneumonia, acute cardiovascular events, and pulmonary embolism also as contributing factors [8,9]. Numerous studies have shown the effects of pre-fracture comorbidities on postoperative complications and mortality [[10], [11], [12], [13], [14]]. One such tool is the Charlson Comorbidity Index (CCI), which is an easy, inexpensive, and quick method for pre-surgical assessment of risk factors. CCI considers major medical comorbidities and demonstrates short-term predictive ability for postoperative complications in hip fracture patients [15]. If hip fracture is left untreated for medical reasons, the one-year mortality would be more than 60 % for these patients [[16], [17], [18]]. Process management is essential for the treatment of hip fractures. Performing surgery within 24 h or 48hr of hospital admission can reduce the mortality rate of patients [19,20]. Treating these patients together with colleagues in geriatric medicine, the so-called orthogeriatric co-management or geriatric trauma unit, resulted in an additional reduction in mortality rates [21].

Several studies reported the outcome of nonagenarian patients with hip fracture receiving operation. However, there are few studies comparing nonagenarian patients who underwent surgery and those who were conservatively treated. Herein, we conducted a retrospective study on patients over 90 years of age who suffered from a hip fracture. This study aimed to compare the risk factors and overall survival between groups that received surgical intervention and conservative treatment in nonagenarian patients.

## 2 Methods

### 2.1 Patient selection

This study was conducted at the Chang Gung Memorial Hospital Keelung branch. The majority of the population in Keelung City is Taiwanese Chinese. Patients aged ≥90 years who visited the emergency department with hip fractures between March 2010 and December 2020 were identified from the trauma database of the hospital. Patients who underwent surgery for hip fractures and those who received conservative treatment were also included. Patients with femoral neck and intertrochanteric fractures were included, but those with periprosthetic, subtrochanteric, or concomitant femoral shaft fractures were excluded. We also excluded hip pathologic fractures, septic hip arthritis, and metabolic or inflammatory bone diseases. The medical records of all identified patients were retrospectively reviewed. Information regarding age, sex, type of fracture, underlying diseases, injury severity score (ISS), and timing and types of surgery were recorded. The study was approved by the Institutional Review Board of Chang Gung Medical Foundation (approval number: 202200600B0) and was waived the informed consents owing to the retrospective nature of this study.

Pre-operative Comorbidity.

Previous studies have shown that CCI predicts the complication rate and survival of patients treated surgically for hip fracture [[22], [23], [24]]. Therefore, we reviewed the medical records of the included patients and identified the underlying diseases listed in the CCI.

### 2.2 Outcomes

The primary outcome was the overall survival of the surgical and non-surgical groups. Overall survival was defined as the time from diagnosis to death. If the patients were alive and attached to follow-up, they were censored at the date of the last follow-up. We also compared the mortality rates at 30 days, 1 year, and 2 years. We also recorded the length of hospital stay, 30-day complication rates, and 30-day readmission rates.

### 2.3 Statistical analysis

Non-normally distributed continuous variables were recorded as medians with interquartile range (IQR), and Mann-Whitney U-tests were used to test for differences. Categorical variables were compared using the chi-squared test or Fisher's exact test.

Sample sizes and power analysis were evaluated by package (powerSurvEpi) from R version 4.1.2 (R Foundation for Statistical Computing, Vienna, Austria) [25]. We used log-rank tests for time-to-event endpoints providing two-sided p values. The Kaplan-Meier curve was created. We used a Cox proportional hazards model to estimate the unadjusted hazard ratios (HR) and 95 % CIs (confidence intervals). Potential clinically relevant covariates were included. Multivariate cox regression model was finally conducted. All statistical analyses, except sample sizes and power analysis, were performed using IBM SPSS Statistics 28 software (IBM Corporation, Armonk, NY, USA).

## 3 Results

### 3.1 Clinical characteristics

**Fig. 1:**
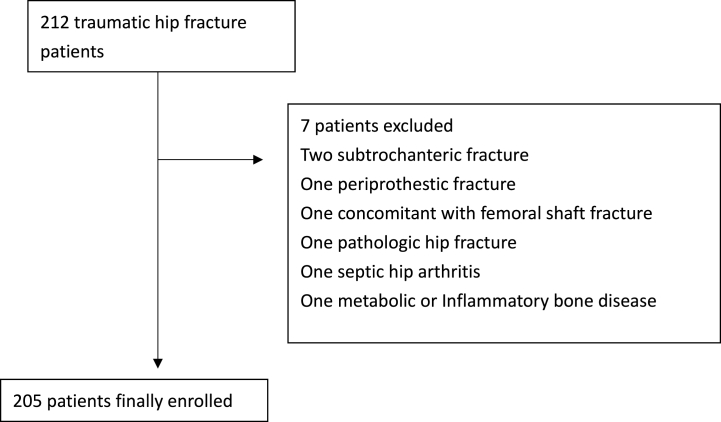
Flow chart of the study.

A total of 173 patients who underwent surgery (OP group) and 32 who received conservative treatment (non-OP group) were included (Fig. 1). The clinical characteristics of the patients are summarized in Table 1. The patients in both groups were predominantly female (OP group, 74 %; non-OP group, 59.4 %), with ages ranging from 90 to 104 years. The median age was 92 years (IQR: 91–94) in the OP and non-OP groups. The cause of fracture in most cases was a fall (99.4 % in the OP group and 100 % in the non-OP group), and only one patient in the OP group (0.7 %) was the pedestrian victim of a traffic accident. There were more intertrochanteric fractures in the OP group than in the non-OP group (64.7 % vs. 31.3 %), and fewer femoral neck fractures in the OP group than in the non-OP group (35.3 % vs. 68.8 %). Hypertension was the most common underlying disease (48.8 %) followed by diabetes mellitus (20.5 %). The median CCI was 1 [IQR: 0–2] in the OP group and 2 [IQR: 1–2] in the non-OP group **(**Table 1**)**. Of the 173 patients who underwent operative management, 70.5 % (n = 122) underwent surgery within 48 h of admission. Dynamic hip screw (DHS) was the most common type of device (n = 68, 39.3 %), followed by cephallomedullary nail (n = 52, 30.1 %), bipolar or total hip replacement (THR) (n = 44, 25.4 %), and cannulated screw (n = 9, 5.2 %) **(**Table 4**)**.

**Table 1:** Demographic data and outcome.

	Total N = 205	OP N = 173	Non-OP N = 32	p
Age (year)	92 [91,94]	92 [91,94]	92 [91,94]	0.578
Gender Male ( %)	58 (28.3)	45 (26.0)	13 (40.6)	0.092
Mechanism ( %) Fall Pedestrian	204 (99.5) 1 (0.5)	172 (99.4) 1 (0.6)	32 (100.0) 0	1
Fracture type ( %) Intertrochanteric Femoral neck	122 (59.5) 83 (40.5)	112 (64.7) 61 (35.3)	10 (31.3) 22 (68.8)	**<0.001**
CCI	1 [0,2]	1 [0,2]	2 [1,2]	0.069
HTN ( %)	100 (48.8)	82 (47.4)	18 (56.3)	0.357
ISS	9 [9,9]	9 [9,9]	9 [9,9]	0.561
Complication 30 days ( %) Pneumonia Sepsis UTI Other	45 (22.0) 20 (9.8) 8 (3.9) 20 (9.8) 11 (5.4)	31 (17.9) 8 (4.6) 6 (3.5) 16 (9.2) 7 (4)	14 (43.8) 12 (37.5) 2 (6.3) 4 (12.5) 4 (12.5)	**0.001** **<0.001** 0.613 0.525 0.073
30-day readmission ( %)	19 (9.3)	12 (6.9)	7 (21.9)	**0.015**
LOS	8 [6,13]	8 [6,13]	8 [1,18.5]	0.551
30-day mortality ( %)	6 (2.9)	3 (1.7)	3 (9.4)	0.050
1-year mortality ( %)	26 (12.7)	21 (12.1)	5 (15.6)	0.568
2-year mortality ( %)	44 (21.5)	36 (20.8)	8 (25)	0.596

OP: Operation, CCI: Charlson Comorbidity Index, HTN: Hypertension, ISS: Injury Severity Score, LOS: Length of Hospital Stay.

### 3.2 Outcome analysis

The median length of hospital stay was 8 days [IQR: 6–13] days in the OP group and 8 days [IQR: 1–18.5] days in the non-OP group, without statistical significance (p = 0.551). More patients in the non-OP group developed complications within 30 days than those in the OP group (43.8 % vs 17.9 %, p = 0.001). Pneumonia was the leading complication in the non-OP group, and the pneumonia rate was much higher than that in the OP group (37.5 % vs. 4.6 %, p < 0.001). In addition, more patients were readmitted within 30 days of discharge in the non-OP group than in the OP group.

A total of 179 patients (151 in the OP group and 28 in the non-OP group) had available records of activities of daily living before and after hip fracture. When the patients could smoothly walk or use a cane before injury, there were 57.9 % bedridden status in the non-OP group after injury, compared to 8 % in the OP group. Among the patients who used a walker before injury, 75 % in the non-OP group were bedridden after injury, compared to 31.3 % in the OP group. There were 66.7 % bedridden status in the non-OP group comparing to 10 % in the OP group after injury when the patients relied on wheelchair for mobility before injury.

Overall, 30-day mortality rates were 1.7 % and 9.4 % in the OP and non-OP groups, respectively. In the OP group, one patient died from pneumonia and two others died from sudden respiratory failure of unknown causes. In the non-OP group, one patient died from pneumonia, one from progressing congestive heart failure, and the last patient had a traumatic subdural hemorrhage at home and died after refusing further surgery. At 1 year after surgery, there were 19 and 5 mortalities in the OP and non-OP groups, respectively; 34 patients from the OP group and 15 patients from the non-OP group were lost to follow-up. Of the 18 patients who died in our hospital between 30 days and 1 year, 9 in the OP group and 2 in the non-OP group died from pneumonia. Between 1 and 5 years, 10 out of 34 documented deaths in the OP group and 1 out of 5 documented deaths in the non-OP group were caused by pneumonia (Table 1).

**Fig. 2:**
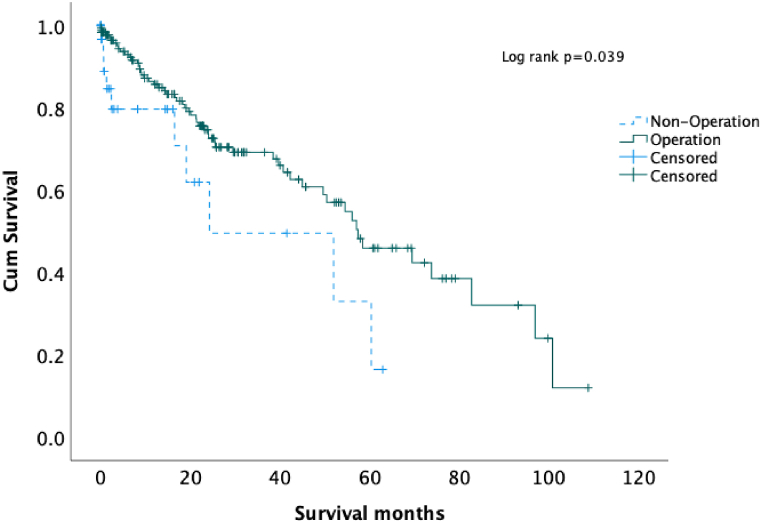
Kaplan-Meier curve for overall survival.

**Fig. 3:**
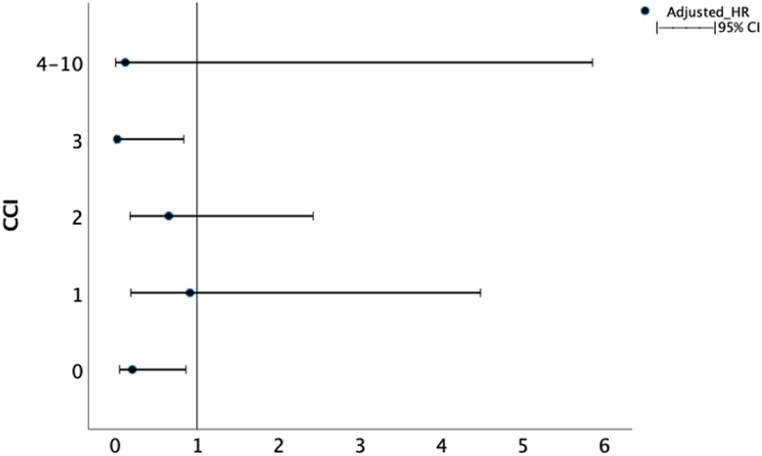
Adjusted HR of operation according to different CCI.

Unadjusted overall survival was greater in patients who underwent operation (median: 57.4 months) than those in the non-OP group (median: 24.3months) (log-rank P = 0.039) (Fig. 2, Table 2). Under multivariate cox regression analysis adjusted other covariates, the risk of death was reduced by surgery (HR = 0.452; 95 % CI: 0.227–0.899; p = 0.024). We also found that CCI was an independent risk factor for poor survival rate (HR = 1.296; 95 % CI: 1.123–1.496; p < 0.001) (Table 3). If patients were stratified by CCI, patients receiving the operation still provided better survival than non-operation in CCI 0 and CCI 3 groups (Fig. 3).

**Table 2:** Overall survival by treatment type.

	Total	Median OS (95 % CI) months	Log-rank P
Operation group	173	57.4 (41.7–73.1)	**0.039**
Non-operative group	32	24.3 (0–57.9)	
Total	205	57.1 (49.4–64.8)	

OS: Overall Survival, CI: Confidence Interval.

**Table 3:** Risk factor for Cox proportional hazards model.

	Unadjusted HR	95 % CI	Unadjusted p	Adjusted HR	95 % CI	Adjusted p
Age	1.049	0.967–1.138	0.25	1.07	0.988–1.16	0.097
Operation	0.495	0.25–0.977	**0.043**	0.452	0.227–0.899	**0.024**
CCI	1.264	1.103–1.45	**<0.001**	1.296	1.123–1.496	**<0.001**
Male gender	0.992	0.565–1.742	0.978			
ISS	1.108	0.852–1.44	0.446			
Fracture type	1.221	0.739–2.016	0.435			

HR: Hazard ratio, CI: Confidence Interval, CCI: Charlson Comorbidity Index, ISS: Injury Severity Score.

**Table 4:** Time to operation after admission.

	<48 h N = 122 (70.5 %)	≥48 h N = 51 (29.5 %)	P Value
Age	92 [91,94]	93 [92,95]	0.096
Gender Male ( %)	27 (22.1)	18 (35.3)	0.072
Fracture type Intertrochanteric Femoral neck	85 (79) 37 (30.3)	27 (52.9) 24 (47.1)	**0.036**
OP method DHS cannulated screw cephallomedullary nail bipolar or THR	46 (37.7) 8 (6.6) 44 (36.1) 24 (19.7)	22 (43.1) 1 (2) 8 (15.7) 20 (39.2)	**0.006**
CCI	1 [0,2]	2 [1,3]	**0.013**
ISS	9 [9,9]	9 [9,9]	0.99
Blood loss ml	150 [100,200]	150 [100,200]	0.869
Complication 30day ( %) Pneumonia UTI Sepsis	19 (15.6) 3 (2.5) 10 (8.2) 4 (3.3)	12 (23.5) 5 (9.8) 6 (11.8) 2 (3.9)	0.213 0.05 0.565 1
30-day readmission ( %)	5 (4.1)	7 (13.7)	**0.043**
LOS (day)	8 [6,11]	12 [7.5,17]	**<0.001**
30-day mortality	2 (1.6)	1 (2)	1
1 year mortality	10 (8.2)	11 (21.6)	**0.014**

DHS: Dynamic Hip Screw, THR: Total Hip Replacement, CCI: Charlson Comorbidity Index, ISS: Injury Severity Score, UTI: Urinary Tract Infection, LOS: Length of Stay.

We also performed a subgroup analysis of the OP group. Patients who underwent surgery within 48 h of admission had lower CCI, higher intertrochanteric fracture rate, lower 30-day pneumonia rate, lower bedridden rate, lower length of stay (LOS), and lower 1 year mortality than those who underwent surgery >48 h after admission **(**Table 4**)**. Kaplan-Meier method was used and revealed that the median overall survival following an operation within 48 h of admission was more than that following an operation after >48 h of admission (73.8 months vs 39.7 months, log rank p = 0.002). After adjusting for several factors including CCI, operation more than 48 h of admission decreased the overall survival compared to operation within 48 h of admission (HR: 2.083; 95 % CI: 1.119–3.875; p = 0.021) (Table 5). CCI remained an independent risk factor for poor survival (HR = 1.195; 95 % CI: 1.015–1.408; p = 0.033).

**Table 5:** Risk factor for survival analysis in operative group.

	HR	95 % CI of HR	p
Age	1.066	0.966–1.177	0.205
Male gender	0.913	0.464–1.797	0.791
Fracture type ( %) Femoral neck	1.216	0.4–3.701	0.731
OP method ( %) DHS cannulated screw cephallomedullary nail bipolar or THR	Reference 0 1.107 0.922	0.49–2.503 0.268–3.172	0.991 0.971 0.806 0.898
Preoperative Hb	0.85	0.711–1.018	0.077
Blood loss	1.002	0.999–1.005	0.174
Time to operation after admission ≥48 h	2.083	1.119–3.875	**0.021**
CCI	1.195	1.015–1.408	**0.033**
ISS	1.095	0.847–1.416	0.487

HR: Hazard ratio, CI: Confidence Interval, DHS: Dynamic Hip Screw, THR: Total Hip Replacement, CCI: Charlson Comorbidity Index, ISS: Injury Severity Score.

## 4 Discussion

This retrospective observational study presented that nonagenarians patients with hip fractures treated with operation may provide better survival than non-operation, especially less concurrent comorbidity.

Patients with hip fractures who underwent surgery had a lower risk of mortality both one and two years after injury [26]. Surgical treatment is recommended for hip fracture. However, considering the underlying morbidity and complication rates, deciding the treatment strategy for extremely old patients with hip fractures has been a challenge for physicians, patients, and caregivers. Overtreatment has been a concern in geriatrics, and surgical intervention with general anesthesia may potentially influence the survival of extremely old patients. In such cases, a thorough discussion of prognosis should be conducted [27].

It has been shown that the life expectancy of 90-year-olds (ranging from 4 to 5 years) is still significantly lower than that of 80-year-olds (ranging from 8 to 10 years), even in high-income countries [28]. Our estimated median survival time (57.4 months) demonstrated a similar result in the OP group with a general nonagenarian population, whereas in the non-OP group, the estimated median survival time (24.3 months) was much shorter. In other words, the shortening of expectancy following hip fracture can be offset by surgery.

In the United States, pneumonia and respiratory failure were the most common 30-day complications and cause of 30-day mortality in elderly patients with hip fractures [29], and our study revealed a similar result. At the same time, the rates of 30-day pneumonia and overall complications were significantly lower in the OP group, which can explain why the OP group had lower 30-day admission and 30-day mortality rates.

The functional status could be partially maintained after the operative treatment. Among patients who were able to walk or use a cane before injury, 57.9 % of the non-OP group were bedridden, comparted with 8 % of the OP group after injury. This finding might explain the lower 30-day pneumonia rate and better overall survival in the OP group.

There are few studies focused on comparing outcomes of hip fractures between operative and non-operative in nonagenarian patients. Ooi et al. arranged a two-year study including 46 operative treatments and 38 non-operative treatments for nonagenarian hip fractures patients [30]. They found surgery provides patients with speeder recovery and shorter admission days. The 1 year-mortality rate in OP group was 30 % compared to non-OP group with 45 %. Authors presumed higher mortality rates in the non-operative group could be due to the poorer pre-injury physical condition of these patients. However, there were no comorbidities data provided.

Liu et al. performed a single center retrospective study for nonagenarian patients with femoral neck fracture and examined 53 operative patients versus 33 non-operative patients [31]. The survival time of the operative group was significantly higher than that of the nonoperative group (median: 53 months vs median: 22 months, p = 0.001). They also found almost half of patients who received surgery will survive more than 5 years. They concluded operation is more likely to improve long-term survival than non-operative treatment. The CCI in non-operative group was higher than operative group without statistically significant. However, they did not analyze the CCI with operation by multivariable analysis.

In our study, the 30-day mortality rates in the OP group are not inferior to those of previous studies on short-term mortality after surgery for hip fracture (1.7 % versus a range from 5.5 to 9.6 %) [9,[31], [32], [33], [34], [35], [36], [37], [38]], and are significantly superior to those of the non-OP group (9.4 %). The estimated median survival times in the OP and non-OP groups were 57.4 and 24.3 months, respectively. The result was similar to the previous study from Liu et al. Overall survival in the OP group was higher than that in the non-OP group. One of the most possible reasons that patients receiving non-OP management could be due to poor comorbidity condition. Therefore, we used multivariable Cox regression analysis to adjust other clinical factors including CCI and ISS, and the operative management was an independent factor that improved survival (HR: 0.427; 95 % CI: 0.207–0.882). In addition, we checked the HR of operative treatment according to different CCI. Patients without comorbidity could benefit from operative treatment. Surgical treatment provides patients with earlier ambulation, better functional status and lower bedridden, lower 30-day pneumonia, and lower readmission rates, which could explain the better overall survival than that in the non-OP group.

We also identified that a higher CCI was a significant risk factor for mortality (HR = 1.3, 95 % CI: 1.115–1.515). Previous studies have shown that CCI is a significant risk factor for complications [24,31] and mortality [38,39] in elderly patients with hip fractures. Our analysis yielded similar results. On the contrary, some studies have indicated that the male sex and fracture type also have significant effects on mortality [38,39], but our data do not yield the same result.

In the subgroup analysis of our OP group, surgery within 48 h could improve overall survival. The result correlates with the American Academy of Orthopedic Surgeons guidelines, which suggest that hip fracture surgery within 48 h of admission is related to better outcomes [40]. This could be related to those patients who underwent surgery within 48 h had a lower 30-day pneumonia rate, a lower bedridden rate, and a lower LOS than those who underwent surgery after >48 h. In the subgroup of 51 patients receiving surgery more than 48 h after admission, 38 (79.2 %) patients were waiting for preoperative examination, which was the main reason for the delayed operation. Besides, 15 (29.4 %) patients were undergoing treatment for underlying medical issues, and 10 (19.6 %) patients experienced delays due to lack of availability of operation rooms or medical staff.

Although our study provides an optimistic view of surgical interventions, we acknowledge several limitations. First, there was a high chance of selection bias between operative and conservative groups. Patients with higher comorbidity would be chosen for the conservative group. Therefore, multivariate cox regression analysis was used to adjust other potential confounders including CCI. We also stratified the patients according to CCI to see the HR of operation. Second, this study was limited by its retrospective nature, and the sample size of the non-operative group was small. However, we used sample size and power analysis to analyze the power was enough. Further multicenter prospective studies should be conducted to establish stronger evidence for future practical guidance. Second, the rate of loss to follow-up was high when the duration was long. Patients who were lost to follow-up died, this could have caused bias. Therefore, we used a survival analysis to manage the censored data to evaluate the overall long-term survival. Finally, the outcomes of geriatric patients are highly influenced by socioeconomic issues and the public healthcare system. A wider survey of the East Asian population should be conducted to better understand the cost effect of intervention on extremely old patients with hip fractures.

## 5 Conclusion

Our study suggests that surgical treatment may provide better overall survival for nonagenarian patients with hip fractures than non-operation treatment, especially patients having less concurrent comorbidities. This results in a lower complication rate including lower pneumonia rate and lower readmission rate.

## Data availability statement

The data supporting the findings in this current study are available from the corresponding author upon reasonable request.

## Additional information

No additional information is available for this paper.

## CRediT authorship contribution statement

**Suo-Hsien Wang:** Writing – original draft. **Chia-Wei Chang:** Writing – original draft, Conceptualization. **Shion-Wei Chai:** Validation, Software. **Ting-Shuo Huang:** Supervision. **Rueyshyang Soong:** Supervision, Resources. **Ngi-Chiong Lau:** Investigation, Data curation. **Chih-Ying Chien:** Writing – review & editing, Supervision, Software, Formal analysis, Data curation, Conceptualization.

## Declaration of competing interest

The authors declare that they have no known competing financial interests or personal relationships that could have appeared to influence the work reported in this paper.

## References

[1] Nikkel L.E., Fox E.J., Black K.P., Davis C., Andersen L., Hollenbeak C.S. (2012). Impact of comorbidities on hospitalization costs following hip fracture. J. Bone Joint Surg. Am..

[2] Braithwaite R.S., Col N.F., Wong J.B. (2003). Estimating hip fracture morbidity, mortality and costs. J. Am. Geriatr. Soc..

[3] Schnell S., Friedman S.M., Mendelson D.A., Bingham K.W., Kates S.L. (Sep 2010). The 1-year mortality of patients treated in a hip fracture program for elders. Geriatr. Orthop. Surg. Rehabil..

[4] Sterling R.S. (2011). Gender and race/ethnicity differences in hip fracture incidence, morbidity, mortality, and function. Clin. Orthop. Relat. Res..

[5] Hu F., Jiang C., Shen J., Tang P., Wang Y. (2012). Preoperative predictors for mortality following hip fracture surgery: a systematic review and meta-analysis. Injury..

[6] Gullberg B., Johnell O., Kanis J.A. (1997). World-wide projections for hip fracture. Osteoporos. Int..

[7] Shao C.J., Hsieh Y.H., Tsai C.H., Lai K.A. (2009). A nationwide seven-year trend of hip fractures in the elderly population of Taiwan. Bone..

[8] Schousboe J.T. (2017). Mortality after osteoporotic fractures: what proportion is caused by fracture and is preventable?. J. Bone Miner. Res..

[9] Roche J.J., Wenn R.T., Sahota O., Moran C.G. (Dec 10 2005). Effect of comorbidities and postoperative complications on mortality after hip fracture in elderly people: prospective observational cohort study. BMJ.

[10] Maggi S., Siviero P., Wetle T. (2010). A multicenter survey on profile of care for hip fracture: predictors of mortality and disability. Osteoporos Int..

[11] Hershkovitz A., Polatov I., Beloosesky Y., Brill S. (Sep-Oct 2010). Factors affecting mortality of frail hip-fractured elderly patients. Arch. Gerontol. Geriatr..

[12] Franzo A., Francescutti C., Simon G. (2005). Risk factors correlated with post-operative mortality for hip fracture surgery in the elderly: a population-based approach. Eur. J. Epidemiol..

[13] Holvik K., Ranhoff A.H., Martinsen M.I., Solheim L.F. (Dec 2010). Predictors of mortality in older hip fracture inpatients admitted to an orthogeriatric unit in oslo, Norway. J. Aging Health.

[14] Soderqvist A., Ekstrom W., Ponzer S. (2009). Prediction of mortality in elderly patients with hip fractures: a two-year prospective study of 1,944 patients. Gerontology.

[15] Burgos E., Gomez-Arnau J.I., Diez R., Munoz L., Fernandez-Guisasola J., Garcia del Valle S. (2008). Predictive value of six risk scores for outcome after surgical repair of hip fracture in elderly patients. Acta. Anaesthesiol. Scand..

[16] Frenkel Rutenberg T., Assaly A., Vitenberg M. (2019). Outcome of non-surgical treatment of proximal femur fractures in the fragile elderly population. Injury..

[17] Shin S., Kim S.H., Park K.K., Kim S.J., Bae J.C., Choi Y.S. (May 26 2020). Effects of anesthesia techniques on outcomes after hip fracture surgery in elderly patients: a prospective, randomized, controlled trial. J. Clin. Med..

[18] Murthy S., Hepner D.L., Cooper Z., Bader A.M., Neuman M.D. (Dec 2015). Controversies in anaesthesia for noncardiac surgery in older adults. Br. J. Anaesth..

[19] Aslan A., Atay T., Aydogan N.H. (2020). Risk factors for mortality and survival rates in elderly patients undergoing hemiarthroplasty for hip fracture. Acta Orthop. Traumatol. Turc..

[20] Hapuarachchi K.S., Ahluwalia R.S., Bowditch M.G. (2014). Neck of femur fractures in the over 90s: a select group of patients who require prompt surgical intervention for optimal results. J. Orthop. Traumatol..

[21] Rapp K., Becker C., Todd C. (2020). The association between orthogeriatric Co-management and mortality following hip fracture. Dtsch Arztebl. Int..

[22] Jiang L., Chou A.C.C., Nadkarni N. (2018). Charlson comorbidity index predicts 5-year survivorship of surgically treated hip fracture patients. Geriatr. Orthop. Surg. Rehabil..

[23] Quach L.H., Jayamaha S., Whitehouse S.L., Crawford R., Pulle C.R., Bell J.J. (2020). Comparison of the Charlson Comorbidity Index with the ASA score for predicting 12-month mortality in acute hip fracture. Injury..

[24] Hasan O., Barkat R., Rabbani A., Rabbani U., Mahmood F., Noordin S. (Oct 2020). Charlson comorbidity index predicts postoperative complications in surgically treated hip fracture patients in a tertiary care hospital: retrospective cohort of 1045 patients. Int. J. Surg..

[25] R Core Team. R (2021).

[26] Tay E. (2016). Hip fractures in the elderly: operative versus nonoperative management. Singapore Med. J..

[27] Smith A.K., Williams B.A., Lo B. (Dec 8 2011). Discussing overall prognosis with the very elderly. N. Engl. J. Med..

[28] Thinggaard M., McGue M., Jeune B., Osler M., Vaupel J.W., Christensen K. (2016). Survival prognosis in very old adults. J. Am. Geriatr. Soc..

[29] Groff H., Kheir M.M., George J., Azboy I., Higuera C.A., Parvizi J. (2020). Causes of in-hospital mortality after hip fractures in the elderly. Hip Int..

[30] Ooi L.H., Wong T.H., Toh C.L., Wong H.P. (2005). Hip fractures in nonagenarians--a study on operative and non-operative management. Injury..

[31] Liu Y., Zhang C.W., Zhao X.D. (2020). Long-term survival of femoral neck fracture patients aged over ninety years: arthroplasty compared with nonoperative treatment. BMC Musculoskelet. Disord..

[32] Lin W.T., Chao C.M., Liu H.C., Li Y.J., Lee W.J., Lai C.C. (2015). Short-term outcomes of hip fractures in patients aged 90 years old and over receiving surgical intervention. PLoS One.

[33] van de Kerkhove M.P., Antheunis P.S., Luitse J.S., Goslings J.C. (2008). Hip fractures in nonagenarians: perioperative mortality and survival. Injury..

[34] Chia P.H., Gualano L., Seevanayagam S., Weinberg L. (2013). Outcomes following fractured neck of femurin an Australian metropolitan teaching hospital. Bone Joint Res.

[35] Gregory J.J., Starks I., Aulakh T., Phillips S.J. (2010). Five-year survival of nonagenerian patients undergoing total hip replacement in the United Kingdom. J.J Bone Joint Surg Br. Sep Bone Joint Surg. Br..

[36] de Leur K., Vroemen J.P., Vos D.I., Elmans L., van der Laan L. (2014). Outcome after osteosynthesis of hip fractures in nonagenarians. Clin. Interv. Aging.

[37] Graver A., Merwin S., Collins L., Kohn N., Goldman A. (2015). Comorbid profile rather than age determines hip fracture mortality in a nonagenarian population. Hss J..

[38] Lin J.C., Liang W.M. (Apr 4 2017). Mortality, readmission, and reoperation after hip fracture in nonagenarians. BMC Muscoskel. Disord..

[39] Knauf T., Bücking B., Bargello M. (2019). Predictors of long-term survival after hip fractures?-5-year results of a prospective study in Germany. Arch. Osteoporos..

[40] Roberts K.C., Brox W.T., Jevsevar D.S., Sevarino K. (2015). Management of hip fractures in the elderly. J. Am. Acad. Orthop. Surg..

